# Dynamic change of calcium-rich compartments during coccolithophore biomineralization

**DOI:** 10.1126/sciadv.adv0618

**Published:** 2025-07-23

**Authors:** Alexander Triccas, Daniel M. Chevrier, Mariana Verezhak, Johannes Ihli, Manuel Guizar-Sicairos, Mirko Holler, André Scheffel, Noriaki Ozaki, Virginie Chamard, Rachel Wood, Tilman A. Grünewald, Fabio Nudelman

**Affiliations:** ^1^ School of Chemistry, University of Edinburgh, Joseph Black Building, David Brewster Road, Edinburgh EH9 3FJ, UK.; ^2^ Université Aix-Marseille, CNRS, CEA, BIAM, UMR7265 Institut de Biosciences and Biotechnologies d’Aix-Marseille, F-13115 Saint-Paul-lez-Durance, France.; ^3^ Photon Science Division, Paul Scherrer Institut, Forschungstrasse 111, 5232 Villigen PSI, Switzerland.; ^4^ University of Oxford, Oxford OX2 6NN, UK.; ^5^ Institute of Physics, École Polytechnique Fédérale de Lausanne (EPFL), 1015 Lausanne, Switzerland.; ^6^ University of Greifswald, Institute of Microbiology, 17489 Greifswald, Germany.; ^7^ Department of Biotechnology, Faculty of Bioresource Sciences, Akita Prefectural University, 241-438 Kaidobata-Nishi, Nakano Shimoshinjo, Akita 010-0195, Japan.; ^8^ Aix-Marseille Univ, CNRS, Centrale Med, Institut Fresnel, Marseille, France.; ^9^ School of GeoSciences, University of Edinburgh, James Hutton Road, Edinburgh EH9 3FE, UK.

## Abstract

Coccolithophores are abundant marine phytoplankton that produce biomineralized calcite scales, called coccoliths, which sequester substantial amounts of carbon and play a substantial role in biogeochemical cycles. However, mechanisms underlying the storage and transport of ions essential for calcification remain unresolved. We used ptychographic x-ray computed tomography under cryogenic conditions to visualize intracellular calcium-rich structures involved in the storage of calcium ions in the coccolithophore species *Chrysotila carterae*. During calcification, we observed a range of structures, from small electron-dense bodies within larger compartments to denser and distributed globular compartments, before returning to small bodies once scale formation is complete. Nanobeam-scanning x-ray fluorescence measurements further revealed that these electron-dense bodies are rich in phosphorus and calcium (molar ratio of ~4:1). The dynamic nature of structures suggests that these bodies are part of the required cellular calcium ion transport pathways, a fundamental process critical for understanding the response of coccolithophores to climate change.

## Teaser

The distribution of intracellular calcium storage compartments in coccolithophores changes as a function of calcification.

## INTRODUCTION

Coccolithophores are marine unicellular algae that are among the most prolific calcifiers in the oceans. They produce polycrystalline scales made of calcite called coccoliths, displaying a remarkable ability to orchestrate the multilevel assembly of nanocrystalline building blocks into a higher-order structure. Coccolith production is of high importance to Earth’s biogeochemical cycle. Their formation is responsible for the sequestration of large amounts of CO_2_ within ocean sediments, forming the largest geological sink of carbon from the ocean/atmosphere reservoir (*1*). The continuous sinking of the scales to the ocean floor not only provides ballast for the transport of organic matter to the deep sea (*2*) but is also important for maintaining the vertical gradient in sea water alkalinity (*3*) and providing important stabilizing feedback in Earth’s climate system (*4*). Despite the significance of coccolithophore biomineralization for our environment, we still know little about the mechanisms governing the calcification and how they can be affected by changes in ocean chemistry caused by anthropogenic activity.

Coccolith production takes place intracellularly, in a specialized compartment called the coccolith vesicle (*5*). Within, calcite crystals nucleate around the edge of an organic template called the base plate to form a ring of rhombohedral crystals called the protococcolith ring (*5*–*7*). Single-crystalline units then extend sideways and outward, mechanically interlocking as they develop. The growth and overall morphology are controlled by coccolith-associated polysaccharides (*8*–*10*), a constrained space for growth (*11*, *12*) and ion gradients (*13*). Once complete, coccoliths, with size in the 1- to 10-μm range, are exocytosed and, together with previously formed scales, form an exoskeleton around the cell called the coccosphere (*14*). The cellular mechanisms that control different aspects of coccolith mineralization are still left to be clarified. One particular area that is not well understood is the mass transport of ions across the cell to the mineralization site. A substantial quantity of calcium ions is required to produce condensed mineral phases, which raises questions on how coccolithophores, as well as eukaryotic cells in general, avoid cytotoxicity. Calcium concentration in the cytosol must be kept around 100 nM to prevent toxicity and eventual cell death (*15*). For calcite to precipitate, concentrations within the coccolith vesicle must be raised to 100 to 200 μM (*16*). Cellular pathways are therefore required to transport calcium ions to satisfy the demands of calcification, without raising cytosolic concentrations to fatal levels.

Several biomineralizing organisms, including corals, foraminifera, and sea urchin larvae, source calcium from the surrounding seawater by endocytosis (*17*–*19*). Once inside the cell, a hydrated amorphous calcium carbonate (ACC) phase forms within intracellular vesicles, which are transported to the mineralization site (*20*). In the case of coccolithophores, calcium enters the cell via ion channels on the plasma membrane, before being loaded into endomembrane compartments (*21*, *22*). For the coccolithophore *Chrysotila carterae*, calcium ions are delivered to the coccolith vesicle in the form of coccolithosomes, which are electron-dense ~25-nm calcium-associated polysaccharide complexes that originate in the Golgi cisternae (*5*, *23*). Once inside the vesicle, they provide calcium ions for nucleating and growing calcite crystals (*6*, *24*).

It is unclear how calcium ions are transported across the cell into the Golgi network in order for coccolithosomes to form. Large intracellular pools of calcium and polyphosphates resembling acidocalcisomes, which are storage organelles within numerous prokaryotic and eukaryotic cells (*25*), were identified in coccolithophores (*26*, *27*). The role of these structures in calcification is still debated. While they have been initially proposed to be part of a calcium storage and transport pathway involved in coccolith formation (*26*, *27*), subsequent fluorescent studies suggest that the composition, their distribution, and calcium content did not change during coccolith production (*28*). These findings led to the hypothesis that these compartments may function more passively as storage organelles to control and equilibrate calcium concentrations inside the cell (*28*). However, calcium-rich structures have mostly been characterized from single snapshots taken across cryogenically or chemically fixed coccolithophore cells or with confocal microscopy techniques that have limited spatial resolution (*26*–*28*). As a result, any dynamic changes that may occur while cells are calcifying could remain undetected.

To investigate the potentially hidden dynamic pathways that transport calcium through the cell during calcification, we determined how calcium-rich structures change in distribution and density during this process. For this, whole cells of the coccolithophore *C. carterae* were imaged using ptychographic x-ray computed tomography under cryogenic conditions (cryoPXCT). X-ray ptychography is a quantitative three-dimensional (3D) microscopy approach, which provides access to the mass density at the submicrometer scale (*29*–*33*), reaching up to 4-nm resolution on radiation-insensitive specimens (*34*). In addition, we used nanobeam-scanning x-ray fluorescence (nXRF) to determine the calcium content of these dense structures and assess their overall elemental composition. We identified that the distribution of calcium ions in intracellular structures changed across the window in which calcification was taking place. These findings will strengthen our overall understanding of the calcium transport pathways used by coccolithophore cells to facilitate the demands of coccolith production.

## RESULTS

### Calcium-rich structures in *C. carterae* cells

**Fig. 1.**
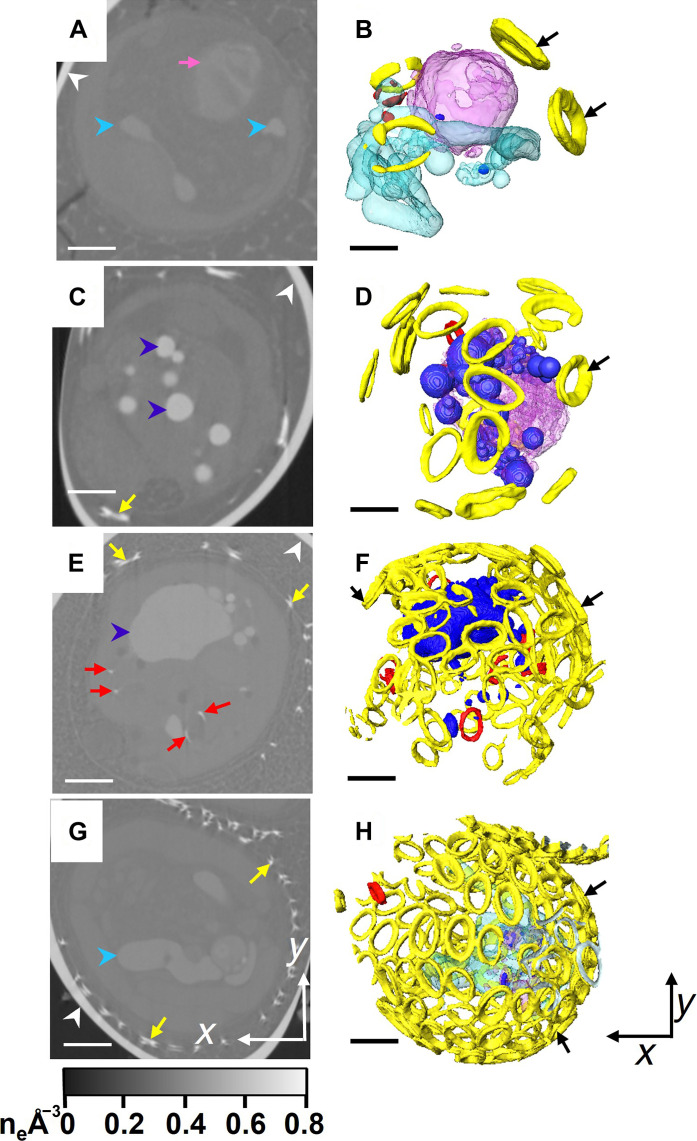
CryoPXCT tomograms of calcifying *C. carterae* cells frozen at different stages of calcification. Left: 2D slice through the volume of individual cells. Right: Respective isosurface-rendered visualization. (**A** and **B**) Stage 1. (**C** and **D**) Stage 2. (**E** and **F**) Stage 3. (**G** and **H**) Stage 4. White arrowheads indicate the glass capillary used to store the sample. Visible are extracellular coccoliths [yellow arrows in (C) to (G) and colored in yellow and indicated by the black arrows in (B), (D), (F), and (H)], intracellular coccoliths [red arrows in (E) and red in (D), (F), and (G)], organelles with electron density between 0.37 and 0.42 n_e_ Å^−3^ [turquoise arrowheads in (A) and (G) and colored in turquoise in (B); pink arrows in (A) and colored pink in (B) and (D)], and structures with electron density above 0.42 n_e_ Å^−3^ [dark blue arrowheads in (C) and (E) and colored dark blue in (B), (D), and (F)]. Scale bars, 2 μm.

Actively calcifying cells of the coccolithophore *C. carterae* were treated with 0.1 M EDTA for 5 min to remove their external coccoliths, before being incubated in low-calcium medium for 24 hours to inhibit mineralization. Calcium was then reintroduced to the medium to restart calcification. After 4, 8, 12, and 24 hours of incubation, cells were loaded into glass capillaries, plunge frozen in liquid ethane, and analyzed using cryoPXCT at −180°C. This synchrotron source–based technique provided 3D reconstructions with quantitative electron density information and spatial resolution in the 10- to 100-nm range (*29*–*31*, *33*). Image segmentation was achieved on the basis of the electron density of the cellular structures, evidencing extracellular and intracellular coccoliths, along with the intracellular organelles and compartments. The longer cells were incubated in calcium-replete medium, more coccoliths were produced, and the coccosphere became more developed. A degree of variability in the rate of formation of the coccosphere was observed at each incubation time, and, therefore, the cells were categorized according to the stages of completeness in the formation of their coccospheres, as follows: stage 1, where less than 20% of the coccosphere was complete; stage 2, where 20 to 40% of the coccosphere was complete; stage 3, with 40 to 60% of the coccosphere complete; and stage 4, which included cells with more than 60% of the coccosphere complete. This is represented in Fig. 1, where an example of one cell at each state of formation is shown. In stage 1, few external coccoliths (1 to 5, up to 5% of the coccosphere complete) were present (in yellow, Fig. 1, A and B, and movie S1). In stage 2, 15 to 20 external coccoliths (15 to 20% of the coccosphere complete) were spread around the cell surface (Fig. 1, C and D, yellow, and movie S2). In stage 3, the coccosphere was ~50% complete, with adjacent coccoliths starting to connect (Fig. 1, E and F, yellow, and movie S3). At these three stages of coccosphere formation, most cells contained intracellular coccoliths (colored as red for sake of clarity in Fig. 1), indicating that calcification was actively taking place within. Last, in stage 4, large parts of the coccosphere were complete (>70%; Fig. 1, G and H, yellow). While a forming coccolith can be observed in the cell shown in Fig. 1 (G and H) (see also movie S4), three of six cells at this stage contained no intracellular mineral structures (movie S5 depicts the reconstructions of all six cells in this stage). In all cases, additional features were visible alongside the intracellular and external coccoliths, including structures with electron density between 0.42 and 0.62 number of electrons (n_e_) Å^−3^ (Fig. 1, dark blue) and organelles and compartments with electron density between 0.37 and 0.40 n_e_ Å^−3^ that were either empty (Fig. 1, turquoise) or containing the denser structures with electron density between 0.42 and 0.62 n_e_ Å^−3^ (Fig. 1, pink).

### Composition of the dense intracellular bodies in *C. carterae*

As the electron density for most metal-free cellular bodies is reported (*35*) to reach up to 0.35 n_e_ Å^−3^, we hypothesize that the structures with values of 0.37 n_e_ Å^−3^ and higher contain heavier elements, with calcium being a likely candidate considering that it is stored inside intracellular compartments (*26*, *27*). To verify this hypothesis, we determined the elemental composition of the dense intracellular structures by nXRF.

**Fig. 2.**
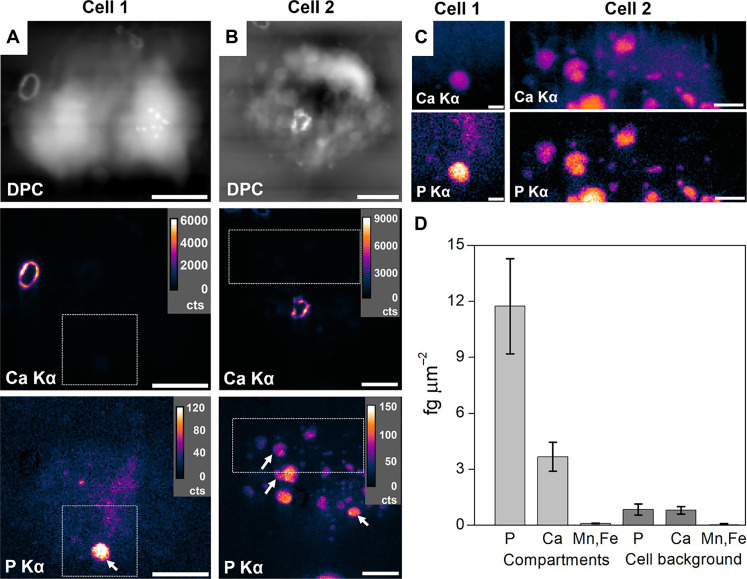
nXRF measurements of dried *C. carterae* cells. DPC and XRF maps of (**A**) cell 1 and (**B**) cell 2 harvested after 4 hours in Ca-replete medium. Ca Kα and P Kα XRF signals are shown with linear-based intensity from detector counts (cts). (**C**) Zoom-in of indicated regions in (A) and (B) with Ca XRF signal now presented in log-based intensity to reveal weaker signals. (**D**) Semiquantitative elemental quantities extracted from dense compartments [indicated with arrows in (A) and (B)] and cell background region. Scale bars, 3 μm [(A) and (B)], 500 nm [(C), cell 1], and 1 μm [(C), cell 2].

*C. carterae* cells were collected after 4 hours in Ca-replete medium, dried on substrate, and then imaged by nXRF (fig. S1 shows representative fitting examples for nXRF datasets). In this approach, the signal is summed along the beam propagation direction, so that the presented image corresponds to an integration of the signal along the cell thickness. Differential phase contrast (DPC) was collected simultaneously with nXRF to provide a reference image of the coccolithophore cell measured (see Materials and Methods). DPC maps of two cells are presented in the first row of Fig. 2 (A and B), both showing one associated coccolith [fig. S2 shows a third cell (cell 3) with several associated coccoliths]. The two subsequent rows are elemental maps of Ca and P. The Ca Kα XRF signal is strongly associated with coccolith materials, while the P Kα XRF signal pertains to intracellular structures including small bodies. Inspecting these bodies further in Fig. 2C (see dashed box regions in Fig. 2, A and B) reveals colocalization of weaker Ca XRF intensities with P. These features are comparable in appearance to the roundish structures in cryoPXCT tomograms (Fig. 1C). Cell 2 (Fig. 2B) contains many more dense compartments than cell 1; it suggests that cell 2 was at a later stage of calcification (e.g., see stage 2 cryoPXCT in Fig. 1, C and D). It is expected that lower electron density features (e.g., 0.37 to 0.40 n_e_ Å^−3^) are less discernible with nXRF and that their structural preservation is likely lost.

The semiquantitative elemental composition (standard reference–calibrated and substrate background– and area-corrected signals) of the denser features and the cell background is presented in table S1 and summarized in Fig. 2D. Most notable is the ~13-fold increase in P and ~4-fold increase in Ca in dense compartments compared to the cell background. Other elements, such as Mn and Fe (figs. S2 and S3), are lower in concentration compared to Ca and P and likely have little contribution to the electron density values measured from cryoPXCT data. In addition to the increase in P and Ca in the compartments, the relative ratio of these quantities changes from a P/Ca of ~1.1 in the cell background to ~3.2. Converting mass to equivalent moles (mole of P / mole of Ca), a ratio of ~4.1 is calculated. Together, nXRF shows that dense compartments in calcifying *C. carterae* cells are rich in P and Ca content.

### Stage 1—Start of coccosphere formation

**Fig. 3.**
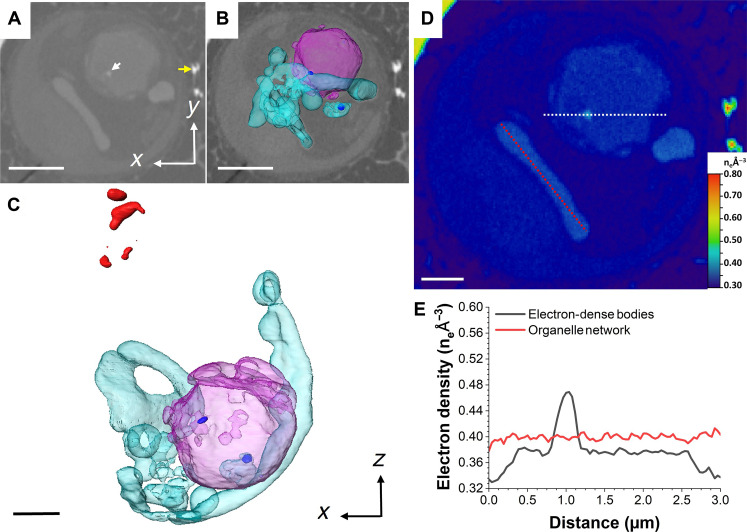
CryoPXCT tomogram of intracellular structures within a *C. carterae* cell at stage 1. (**A**) 2D slice through the volume of the cell. White arrow, electron dense body; yellow arrow, mature coccolith on the surface of the cell. The 3D isosurface-rendered visualization of the intracellular electron-dense structures is shown overlaid on the 2D slice (**B**) and in more detail in (**C**). Visible are a spherical compartment (pink) with an electron density of ~0.37 n_e_ Å^−3^ containing bodies with an electron density above 0.43 n_e_ Å^−3^ (dark blue), an organellar network with an electron density of 0.40 n_e_ Å^−3^ (turquoise), and potential calcite deposits of forming coccoliths with an electron density above 0.70 n_e_ Å^−3^ (red). (**D**) Electron density map of (A). (**E**) Electron density line profiles of the areas marked by the white and red dotted lines in (D). Scale bars, 2 μm [(A) and (B)] and 1 μm [(C) and (D)].

We hypothesize that the Ca-P–rich bodies identified by nXRF correspond to the electron-dense structures imaged by cryoPXCT in cells at stage 2. They have comparable sizes, and the higher electron density is indicative of the presence of heavier elements, of which calcium is the most likely candidate, given that it is known to be stored by the cells. We therefore analyzed in detail the dynamics of the electron-dense structures as a function of development stage of the coccosphere using cryoPXCT. One cell at stage 1 is shown in Fig. 3 and movie S1. A 2D cross section across the cell (Fig. 3A) and 3D visualization (Fig. 3, B and C) highlight the presence of an organellar network (turquoise) with a uniform electron density of 0.40 n_e_ Å^−3^, suggesting that Ca^2+^ ions were homogeneously distributed inside (Fig. 3, D and E). We postulate that this organellar network corresponds to the endoplasmic reticulum, based on its morphology and its demonstrated capacity to sequester substantial amounts of calcium (*36*). However, given the limited knowledge on the morphology and plasticity of organelles in *C. carterae*, this network could also correspond to mitochondria or Golgi cisternae, both of which are also known to sequester calcium. Juxtaposed to this organellar network was one spherical compartment ~3 μm in diameter, with an electron density of 0.38 n_e_ Å^−3^. This compartment contained two ~350-nm bodies where electron density reached 0.47 n_e_ Å^−3^, which, together with the nXRF data, indicates they are Ca-P–rich bodies (white arrow in Fig. 3A, dark blue in Fig. 3, B and C, and electron density shown in Fig. 3, D and E). We hypothesize that this organelle is an ion-rich compartment, which stores Ca-P–rich bodies and has similar morphology (*37*); however, we cannot discard the possibility that it corresponds to the cell vacuole (*37*). Structures with an electron density of ~0.70 to 0.75 n_e_ Å^−3^ (Fig. 3C, in red, and fig. S4) were also present. These values are more consistent with that of a mineral phase (*38*), indicating that it is composed of calcium carbonate (fig. S4) and may represent the onset of calcite deposition during the formation of the protococcolith or a malformed coccolith. A second cell at stage 1, with only one external and no intracellular coccoliths, displayed a similar distribution of calcium-rich structures, only with a larger number of dense bodies of an electron density of 0.48 n_e_ Å^−3^ contained within compartments of an electron density of 0.37 n_e_ Å^−3^ (fig. S5 and movie S6).

### Stage 2—Early stage of coccosphere formation

**Fig. 4.**
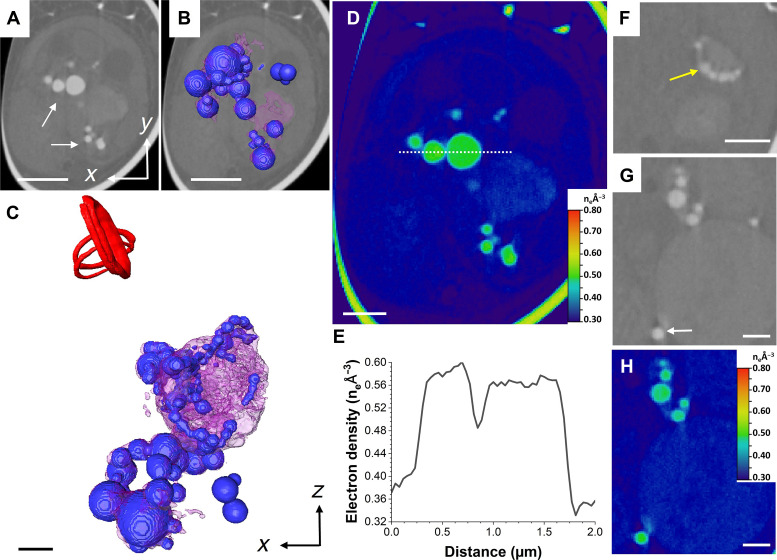
CryoPXCT tomogram of intracellular structures within a *C. carterae* cell at stage 2. (**A**) 2D slice through the volume of the cell. The 3D isosurface-rendered visualization of all electron-dense structures is shown overlaid on the 2D slice (**B**) and in more detail in (**C**). Visible are dense bodies with an electron density between 0.56 and 0.60 n_e_ Å^−3^ distributed across the cell [arrow in (A) and dark blue in (B) and (C)], intracellular structures with an electron density of 0.37 n_e_ Å^−3^ (pink), and forming intracellular coccoliths (red). (**D**) Electron density map of (A). (**E**) Electron density line profile of the area marked by the white dotted line in (D). (**F** and **G**) Two 2D slices from different areas of the cell showing in higher detail electron-dense bodies [yellow arrow in (F) and white arrow in (G)] inside the compartment depicted in pink in (C). (**H**) Electron density map of (G). Scale bars, 2 μm [(A) and (B)] and 1 μm [(C) to (H)].

Two cells were analyzed at stage 2 displaying multiple external coccoliths, as well as intracellular ones being formed. The first cell is depicted in Figs. 1 (C and D) and 4 and movie S2, and the second cell is shown in fig. S6 and movie S7. The morphology and distribution of the electron-dense Ca-P–rich bodies were vastly different compared to those present at stage 1: They were substantially more abundant, had globular shapes, and were clustered into a network-like structure (arrows in Fig. 4A and blue in Fig. 4, B and C), resembling those described by cryo–x-ray tomography (*26*). The size of the bodies varied, with some as small as 350 nm, while others reached up to 1.7 μm. Their electron densities had also substantially risen compared to stage 1, with maximum values between 0.57 and 0.61 n_e_ Å^−3^ (Fig. 4, D and E). When bodies were localized inside lower-density structures, they were always close to the periphery (Fig. 4F, yellow arrow), in some cases seemingly budding off (Fig. 4G, white arrow). Electron density values in these bodies were within the same range as those freely distributed (Fig. 4H), and we speculate that they could have been produced within the larger organelles, before budding off. While the second cell displayed similar globular structures, they were lower in number when compared to the first cell at this stage (fig. S6 and movie S7). The organellar network with uniform electron density was not distinguishable at this stage. A possible cause is the depletion of its Ca^2+^ content, decreasing the electron density and making it indistinguishable from the neighboring intracellular structures and organelles with an electron density up to 0.35 n_e_ Å^−3^.

### Stage 3—Midstage of coccosphere formation

**Fig. 5.**
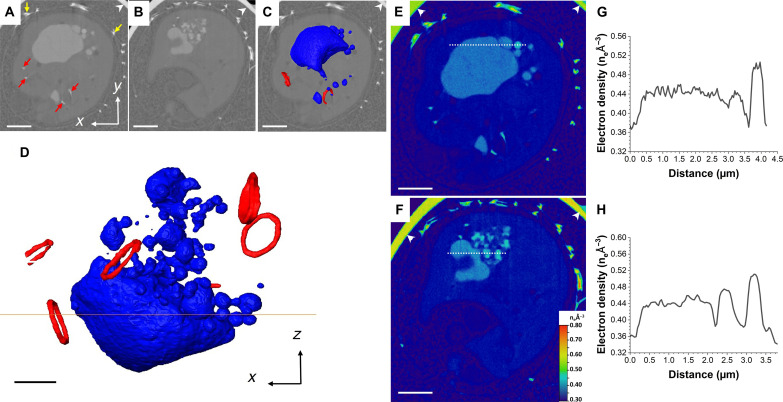
CryoPXCT tomogram of intracellular structures within a *C. carterae* cell at stage 3. (**A** and **B**) 2D slices through the volume of the cell. Red arrows, forming coccoliths; yellow arrows, mature coccoliths on the surface of the cell. The 3D isosurface-rendered visualization of the intracellular electron-dense structures overlaid on the 2D slice from (A) is shown in (**C**) and in more detail in (**D**). Visible are dense bodies with an electron density between 0.44 and 0.52 n_e_ Å^−3^ (dark blue) and nascent intracellular coccoliths (red). (**E** and **F**) Electron density maps of (A) and (B), respectively. (**G**) Electron density line profile of the area marked by the white dotted line in (E). (**H**) Electron density line profile of the area marked by the white dotted line in (F). Scale bars, 2 μm [(A) to (C), (E), and (F)] and 1 μm (D). Arrowheads indicate the wall of the glass capillary used to store the sample.

Two cells with approximately half of the coccosphere complete were classified to be at stage 3 (Figs. 1, E and F, and 5A, yellow arrows; movie S3; and additional cell at this stage in fig. S7 and movie S8). Intracellular coccoliths at various stages of maturity were present (red arrows in Fig. 5A and segmented in red in Fig. 5, C and D; fig. S7, B and C). A substantial portion of the cell was dominated by one large electron-dense body, around 2 to 3 μm in diameter at its thickest (Fig. 5, A, C, and D, and fig. S7). Electron density was consistent across the body, around 0.44 n_e_ Å^−3^ (Fig. 5, E and G, and fig. S7, D and E). In addition, several smaller electron-dense bodies ranging in size between 160 and 900 nm were present, seemingly extending away from the larger body in an extended network (Fig. 5, D and F, and fig. S7, A to C). Their electron density varied, with values either being 0.44 n_e_ Å^−3^, the same as the larger body, or slightly higher, around 0.52 n_e_ Å^−3^ (Fig. 5, E to H). Compared to those in stage 2 (Fig. 4), the electron-dense bodies in the cells at stage 3 had lower electron density values and less defined morphologies. Given the morphological similarity of the large body to the organelle network present at stage 1, its appearance signifies the reformation of the disperse calcium stores, with the smaller dense bodies coalescing to form the larger structure.

### Stage 4—Final stage of coccosphere formation

**Fig. 6.**
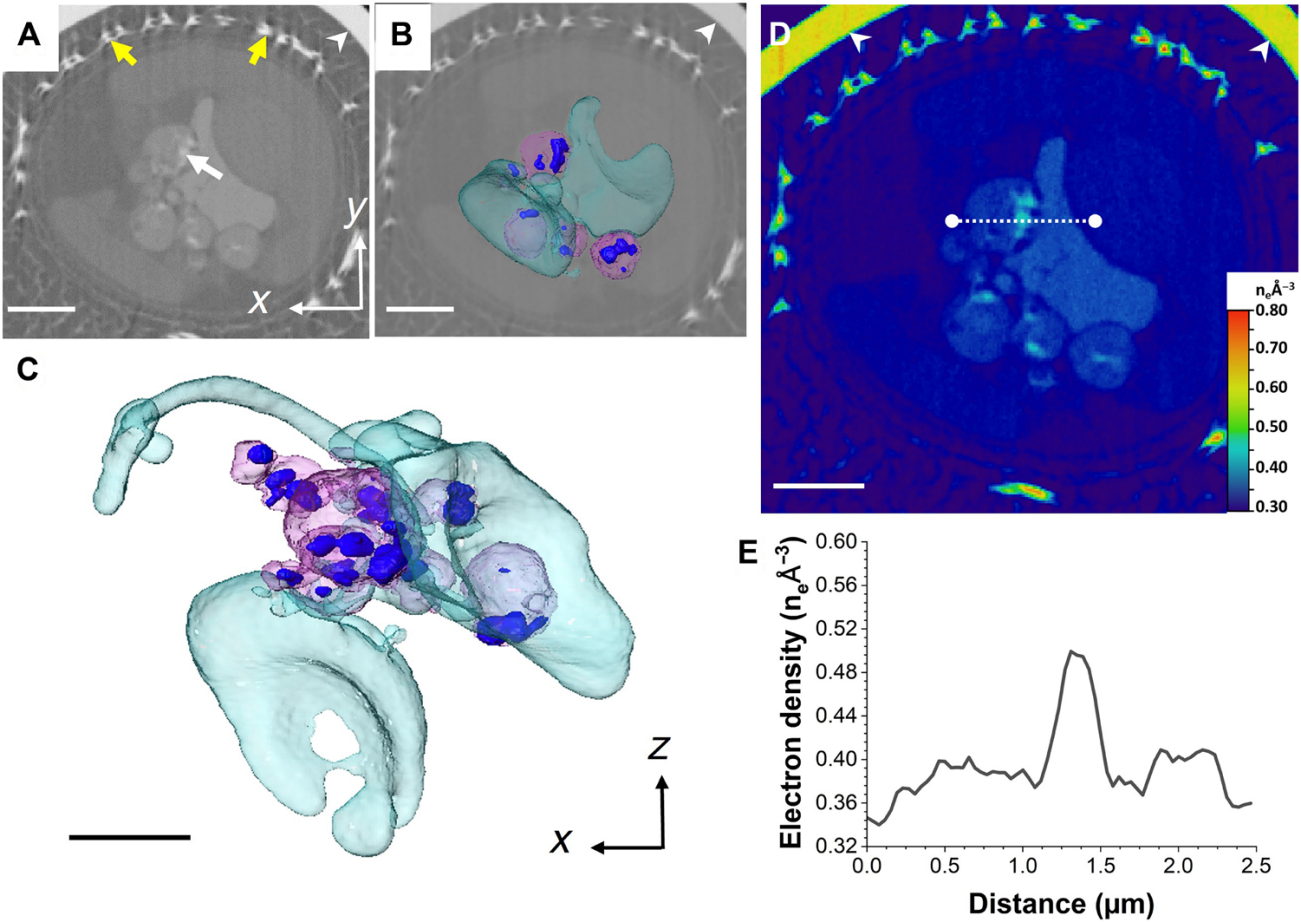
CryoPXCT tomogram of intracellular structures within a *C. carterae* cell after 24 hours in calcium-replete medium. (**A**) 2D slice through the volume of the cell. White arrows, dense bodies with an electron density around 0.50 n_e_ Å^−3^; yellow arrows, extracellular mature coccoliths. The 3D isosurface-rendered visualization of the intracellular electron-dense structures is shown overlaid on the 2D slice (**B**) and in more detail in (**C**). Visible are a spherical compartment (pink) with an electron density of ~0.37 n_e_ Å^−3^ containing bodies with an electron density above 0.43 n_e_ Å^−3^ (dark blue) and an organelle network with an electron density of 0.40 n_e_ Å^−3^ (turquoise). (**D**) Electron density map of (A). (**E**) Electron density line profiles of the areas marked by the white dotted line in (D). Scale bars, 2 μm [(A) to (C)] and 1 μm (D). Arrowheads indicate the wall of the glass capillary used to store the sample.

Six cells were analyzed at stage 4, where large sections of the coccosphere are completed. Intracellular coccoliths were only present in three cells, indicating that calcification may have been slowing down at this time point (*39*). One representative cell where no intracellular coccoliths were present, along with the corresponding 3D visualization, is shown in Fig. 6 and movie S9. Extracellular mature coccoliths are visible (Fig. 6A, yellow arrows), and several 250-nm– to 500-nm–sized Ca-P–rich bodies with an electron densities of 0.49 to 0.51 n_e_ Å^−3^ were present (white arrow in Fig. 6A, dark blue in Fig. 6, B and C, and electron density displayed in Fig. 6, D and E), notably bound within larger compartments with an electron densities of 0.37 to 0.38 n_e_ Å^−3^ [Fig. 6, B and C (in pink), D, and E] In addition, endomembrane compartments with consistent electron density values of 0.40 n_e_ Å^−3^ could be observed [Fig. 6, B and C (in turquoise), D, and E]. Overall, the size, morphology, and distribution of structures with an electron density above 0.37 n_e_ Å^−3^ were similar to those observed in the cells at stage 1 (Fig. 3). A similar distribution of intracellular structures and Ca-P–rich bodies was present in other cells imaged at the same time point, both with and without intracellular coccoliths (fig. S8 and movies S4 and S5).

On the basis of the electron density distribution in intracellular compartments during the various stages of coccosphere formation, we infer that calcium ions were present in intracellular structures in two distinct forms throughout the period when cells were actively calcifying. At the start (stage 1) and toward the end of the formation of the coccosphere (stage 4), calcium ions were dispersed in an organellar network (turquoise in Figs. 3 and 6) and concentrated in small bodies contained within larger compartments. Between these time points, calcium storage switched to being heavily concentrated exclusively inside globular, electron-dense bodies. This transition indicates that ion concentrations can be dynamically altered in the calcium-rich structures to fulfil the demands of coccosphere formation.

## DISCUSSION

The changes in the morphology, intracellular distribution, and electron density of the calcium-rich bodies and compartments during coccosphere formation reported in this study demonstrate the dynamic nature of these structures in the coccolithophore *C. carterae*. At the start of coccosphere formation—when the first coccoliths are being produced—calcium ions were homogeneously dispersed in the organellar network and concentrated in ~300-nm–sized Ca-P–rich bodies located within spherical compartments. As the formation of the coccosphere proceeded, the Ca-P–rich bodies increased in size, electron density, and number, resembling those reported in *C. carterae* cells in previous studies (*26*, *28*). The calcium-rich organellar network was no longer distinguishable, indicating that it had electron density values similar to those of the adjacent cellular structures, likely because of decreased calcium ion content. As the coccosphere neared completion, the distribution of dense bodies resembled the state of the earliest stage. We want to point out that for stages 1 to 3, only two cells were measured per stage. Although they displayed similar distributions of their calcium-rich bodies, a larger sample size, together with additional experiments manipulating the extracellular calcium concentration, is suggested to obtain a more detailed picture of this dynamics.

**Fig. 7.**
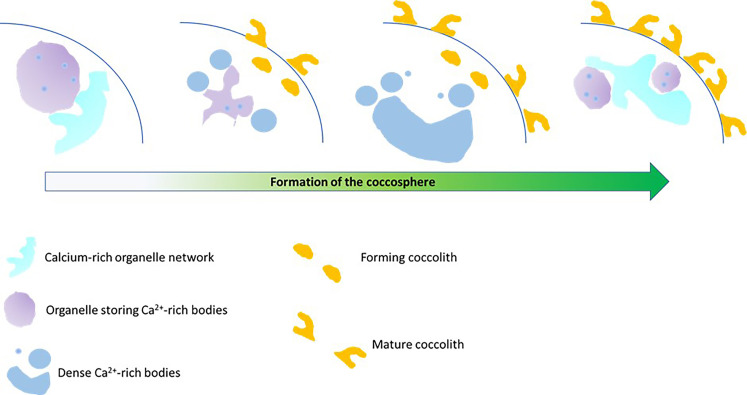
Dynamics of calcium-rich compartments during coccosphere formation in *C. carterae*. Schematic of the proposed changes in the calcium storage compartments of coccolithophores during the formation of the coccosphere.

For the cryoPXCT measurements, individual capillaries were prepared containing cells that were harvested after spending different periods in calcium-replete medium: 4, 8, 12, and 24 hours. Although the different time points help to create different stages of development, each analyzed cell was categorized separately depending in the completeness of the coccosphere. Notably, the capillaries frozen after 8 and 12 hours contained cells at different stages of formation of the coccosphere: One cell at stage 1 (fig. S5) and one at stage 2 (Fig. 4) were found after 8 hours, and one cell at stage 2 (fig. S6) and two cells at stage 3 (Fig. 5 and fig. S7) were present after 12 hours. These observations show that each cell likely started calcifying at different rates after being transferred from calcium-deplete to calcium-replete medium. The distribution of calcium ions between the organellar network and Ca-P–dense bodies was related to the activity of the formation of the coccosphere, rather than the time spent in calcium-replete medium. Therefore, we attribute the distribution of calcium in these structures, as well as their sizes and morphologies, to not be primarily influenced by external ion concentrations. Instead, we suggest that calcium distribution across the organellar network and Ca-P–rich bodies correlates to the calcification process, namely, the mobilization and transport of calcium ions to the coccolith vesicle for coccolith formation. Calcium ions enter the cell via voltage-gated channels (*21*, *22*), where they are proposed to be partitioned directly into endomembrane-derived organelles, to avoid cytosolic concentrations interfering with calcium signaling (*40*). The association of the calcium-rich organellar network with the 1- to 2-μm spherical compartments containing the dense Ca-P–rich bodies suggests a connection between these two organelles. We propose that calcium ions are transported from this organelle to the spherical compartments where they form complexes with polyphosphates (*27*, *41*) to create dense bodies with a P-to-Ca molar ratio of ~4:1, according to our nXRF measurements, enabling high-concentration storage of calcium ions. The dynamics between these two organelles changes as a function of coccosphere formation, which we hypothesize to be in response to the high demand for calcium ions for coccolith formation. In this case, the Ca-P–rich bodies increase in size and number, consuming calcium ions stored in the organellar network, from where they are further mobilized for coccolith formation (*41*). As the coccosphere nears completion, coccolith production slows down, decreasing the demand for calcium ions, which accumulate again in the organellar network. This model is summarized in Fig. 7.

It has been suggested that calcium-rich compartments and Ca-P–rich dense bodies are present in coccolithophores independent of calcification activity, possibly providing an alternate function to the cell (*28*). Spectroscopic studies, however, indicate that ions from the dense bodies become incorporated into the coccolith crystal lattice, which requires a mechanism to transport calcium from the Ca-P–rich bodies into the coccolith vesicle (*41*). Our study shows that the dense bodies fluctuate in electron density and number across coccosphere development, supporting the hypothesis that they alter their ion capacity and function as dynamic calcium reservoirs in response to the demands of biomineral formation (*27*). It is unknown, however, how this transfer occurs. In *C. carterae*, transport of calcium ions to the coccolith vesicle occurs in the form of coccolithosomes, which are ~25-nm calcium complexes that originate in the Golgi complex (*23*, *24*). This transport mechanism implies that calcium ions must first be translocated from the dense bodies to the Golgi. As the cisternae could not be visualized in our cryoPXCT tomograms, we cannot determine how close this organelle approaches the dense bodies.

Because of their resemblance to acidocalcisomes, it appears that calcium-rich pools existed before coccolithophore calcification and may have been adapted to store and transport the large quantities of calcium to facilitate mineral formation. Intracellular calcium pools are not a common feature of extracellular biomineralization processes, being absent in most mineralizing organisms, with ions instead transported alongside carbonate in ACC-containing vesicles derived from the endocytosis of seawater (*18*, *42*). Calcium-rich pools have been reported in dinoflagellates, which, similar to coccolithophores, transport calcium ions into the cell via gated channels (*43*). In dinoflagellates, the calcium-rich bodies are generally associated with extending the mineral front, similar to the ACC-vesicles in other biomineralizing organisms (*19*, *20*, *43*). It is possible that alternate transport pathways may have developed in coccolithophores as a result of crystallization occurring in an intracellular environment. Furthermore, several undetected pathways for ion delivery might exist, varying from species to species, with possible influence from the external cellular environmental conditions.

In conclusion, the findings reported in this study reveal insights into the role of intracellular calcium-rich structures in coccolithophore calcification. By characterizing the calcium distributions during *C. carterae* calcification, we have determined that calcium-storage compartments and Ca-P–rich bodies act as dynamic reservoirs, which are able to change the quantity of ions contained within. These observations indicate that these structures are integral components of the transport pathways responsible for shuttling calcium ions across the cell during calcification. They therefore play an important role in the storage and mobilization of these ions without posing a risk of cytotoxicity.

## MATERIALS AND METHODS

### Culturing

Cultures of *C. carterae* (944/6) were obtained from the Culture Collection of Algae and Protozoa, Oban, Scotland. Cultures were incubated at 18°C on a 12-hour:12-hour light-dark cycle. Cells were grown in natural seawater from St Abbs Marine Station supplemented with Guillard’s (F/2) Marine Water Enrichment Solution (Sigma-Aldrich) and with a penicillin/streptomycin/neomycin antibiotics solution (Thermo Fisher Scientific BioReagents). Cultures were maintained by subculturing 1 ml of culture into 30 ml of fresh medium every 2 weeks. Cell growth was measured using a Leica optical microscope and Neubauer hemocytometer.

### Cryo–ptychographic nanotomography

#### *Preparation of* C. carterae *cells*

*C. carterae* cells were prepared by decalcifying 5 ml of culture in its exponential phase (5 days after subculturing) with 0.1 M EDTA (pH 8.0) to remove external coccoliths. Cells were washed twice in culture medium to remove residual EDTA and then left overnight in low-calcium (100 μM) Aquil artificial seawater to prevent further coccolith growth and recalibrate calcification. A 0.1 M CaCl_2_·2H_2_O (100 μl) solution was then added to raise [Ca^2+^] back to seawater levels (10 mM) and left for 24 hours to provide sufficient time for enough coccoliths to form to fill the entire coccosphere. Cells were in 1 ml of aliquots after 4, 8, 12, and 24 hours in calcium-replete medium, centrifuged at 1500*g*, and concentrated to a ~50-μl volume.

#### Plunge freezing

Capillaries with 10-μm–wide tips were produced using a Flaming-Brown capillary puller (P-2000, Sutter Instruments) and mounted in OMNY pins (*44*) with ultraviolet resin. The concentrated solution of cells at each calcifying time point was loaded into the capillary tips from the accessible backside. The cells were then further sedimented into the very tip of the capillary by a small settling period or by mild centrifugation (1120*g* for 240 s). The capillaries were rapidly plunge frozen in liquid ethane using a vitrification robot (FEI Vitrobot Mark VI). The sample chamber was maintained at 21°C and 100% humidity. Particular care was taken to minimize the time lag between sample preparation and sample freezing. The samples were stored at cryogenic temperature under liquid nitrogen and transferred to the beamline using a transport dewar to ensure a continuous cryogenic cold chain, which was kept up until the samples were loaded into the OMNY instrument (*44*).

#### Imaging parameters

PXCT is a coherent diffractive imaging technique that provides volumetric quantitative electron density of the sample with high spatial resolution (*29*). It relies on illuminating the sample with a coherent, spatially confined x-ray beam. The sample is scanned through the beam, ensuring that the scanning step size is substantially smaller than the illumination footprint. At each point, an intensity diffraction pattern is collected. Through iterative image reconstruction algorithms (*45*), a projection image of the x-ray sample scattering function is obtained, containing phase-shift and absorption information integrated along the beam path, i.e., the sample thickness. By rotating the sample and repeating the ptychography acquisition, a full tomographic dataset is obtained, from which the electron density distribution within the sample can be retrieved in 3D (*30*). The spatial resolution via ptychography is decoupled from the beam size and step size and is ultimately determined by the largest angle at which diffraction signal can be measured with good signal-to-noise ratio and the accuracy of the sample positioning. PXCT measurements were carried out at the coherent small-angle x-ray scattering (cSAXS) beamline (X12SA), Swiss Light Source, Paul Scherrer Institute, Switzerland with the OMNY instrument (*46*). Ptychographic scans were performed at a photon energy of 6.2 keV under cryogenic conditions at 90 K. An Eiger 1.5 M detector, placed 7.2 m downstream of the sample, was used to collect far-field intensity patterns at each scan point. The same ptychography scan was performed at various equiangular orientations, rotating the sample about the vertical axis in the range between 0 and 180°. The exact imaging parameters of each tomogram are listed in table S2.

The ptychographic reconstructions were carried using 300 iterations of the difference-map algorithm (*47*), followed by 300 iterations maximum likelihood refinement (*47*), resulting in a reconstructed pixel size of 38.5 nm (fig. S9) (*45*). The projections were further processed and aligned using in-house scripts (*48*). To provide a first estimate of the spatial resolution a classical approach was used. The datasets were split angularly into two separately reconstructed datasets and compared using the Fourier shell correlation (FSC) approach (*49*). In this way, the half-bit half-period resolution was estimated as ~53 nm, for a detailed discussion on the resolution of PXCT in the datasets presented, see Supplementary Discussion on the Spatial Resolution of PXCT. The corresponding FSC curves are presented in fig. S9, and the detailed FSC figures are tabulated in table S2.

Some residual artifacts, likely caused either from misalignment or radiation-induced effects, were observed in the tomogram of the cells at stage 4 (movies S4, S5, and S9). Using the glass capillary as a reference, its morphology and electron density were comparable with those of the other tomograms (0.64 to 0.66 n_e_ Å^−3^). We therefore considered that these artifacts did not substantially affect the electron density or the morphology of the intracellular structures in the coccoliths in this sample.

#### Segmentation of tomograms

The reconstructed cryoPXCT tomograms gave quantitative electron density values (in number of electrons per cubic angstrom) (*29*). All segmentation was carried out on Avizo software (Thermo Fisher Scientific). For segmentation of electron-dense structures, a minimum electron density threshold value of 0.37 n_e_ Å^−3^ was chosen to determine where calcium ions were located. Localized and well-defined regions of an electron density above 0.43 n_e_ Å^−3^ were often present within the larger compartments with electron density values between 0.37 and 0.43 n_e_ Å^−3^. Those well-defined, denser regions were segmented separately from the compartment that contained them.

### nXRF imaging

Measurements were conducted at I14 x-ray nanoprobe beamline of the Diamond Light Source (*50*). Samples were dried on Si_3_N_4_ membranes and measured at ambient pressure and temperature. An incident photon energy of 8 keV was used with a focused x-ray beam size of ~60 nm (full width at half maximum). A raster scanning step size of 50 or 100 nm was used with a dwell time of 50 ms to collect high-resolution XRF maps. XRF from the sample was collected in front of the sample using a four-element silicon drift detector (RaySpec, UK). A photon-counting Merlin detector (Quantum Detectors, UK) was used in transmission configuration to collect scattered x-rays for DPC images. For semiquantitative analysis of elemental composition, an AXO XRF standard (AXO-DRESDEN GmbH) was measured using identical scanning parameters to coccolithophore samples. The summed XRF spectrum of the AXO standard was used to calibrate the elemental concentrations of P, S, Ca, Mn, and Fe for semiquantitative analysis with reported quantities in femtograms per square micrometer. PyMCA 5.9.2 software was used to energy-calibrate XRF spectra, subtract background spectra, fit XRF sum spectra, calibrate XRF counts to elemental quantities with AXO XRF standard material, and export XRF maps for individual emission lines (e.g., Ca Kα). ImageJ was used to further render XRF maps.

## Supplementary Materials

### The PDF file includes:

Supplementary Discussion Figs. S1 to S9 Tables S1 and S2 Legends for movies S1 to S9
